# Non-IgE-mediated food allergy? A case report

**DOI:** 10.1186/2045-7022-5-S3-P63

**Published:** 2015-03-30

**Authors:** Rosario González-Mendiola, Maria Luisa Galve Martin, Ana Rosa Alcorta Valle, Javier Dionicio Elera, Mariana Herranz Mañas, Irene Carrasco García, Alicia Moral Morales, Herminio Blanco Moratiel, Maria Luisa Gonzalez Morales, Jose Julio Laguna Martínez

**Affiliations:** 1 Allergy Unit, Hospital Central de la Cruz Roja, Madrid, Spain; 2 Department of Gastroenterology, Hospital Central de la Cruz Roja, Madrid, Spain; 3 Department of Anatomical Pathology, Hospital Central de la Cruz Roja, Madrid, Spain

## Background

A 23-year-old woman without personal or family history of atopy presented oral itching, tongue swelling, retrosternal pain and dysphagia when she ate peach, watermelon melon, banana, kiwi, pineapple, strawberry, walnut and hazelnut. She usually eats apple, pear, plum, grapes, peanuts, sunflower seeds and almond. She is asymptomatic on contact with rubber latex objects.

## Method

We performed skin prick tests (SPT) with commercial extracts of common aeroallergens, fruits, nuts, latex, profilin (Pho d 2), polcalcin-enriched date palm, peach LTP (Pru p 3), prick by prick with fresh fruits, specific IgE to pollens, fruits, nuts and latex, epicutaneous food skin tests and simple blind oral provocation food tests. An upper endoscopy with biopsy was also carried out.

## Results

All SPTs yielded negative results. IgE was <0.35 KU/L to all the allergens tested. Epicutaneous tests were negative as well. No pathological findings were found in the upper endoscopy or biopsies performed. Simple blind oral provocation test with pineapple was positive, with immediate report of oral itching, tongue edema, cough and dysphagia. The patient refused to continue the allergologic study.

## Conclusion

We present a case of oral itching, lingual edema and dysphagia related to the ingestion of several fruits and nuts with a completely negative allergologic study except for a positive oral challenge test with pineapple. The reaction appears to be non-IgE mediated. The avoidance of these foods keeps the patient asymptomatic, tolerating the ingestion of other fruits and nuts.

The patient refused to undergo a new upper endoscopy after the oral provocation test. This might have confirmed suspected eosinophilic esophagitis. Our patient had avoided the foods that triggered her symptoms for a long time. This might be reason why the upper endoscopy was normal.

## Consent

Written informed consent was obtained from the patient for publication of this abstract and any accompanying images. A copy of the written consent is available for review by the Editor of this journal.

